# Use of hemagglutinin and neuraminidase amplicon-based high-throughput sequencing with variant analysis to detect co-infection and resolve identical consensus sequences of seasonal influenza in a university setting

**DOI:** 10.1186/s12879-021-06526-5

**Published:** 2021-08-13

**Authors:** Temitope O. C. Faleye, Deborah Adams, Sangeet Adhikari, Helen Sandrolini, Rolf U. Halden, Arvind Varsani, Matthew Scotch

**Affiliations:** 1 Biodesign Center for Environmental Health Engineering, Biodesign Institute, Arizona State University, Tempe, AZ 85287 USA; 2 Biodesign Center for Personalized Diagnostics, Biodesign Institute, Arizona State University, Tempe, AZ 85287 USA; 3 School of Sustainable Engineering and the Built Environment, Arizona State University, Tempe, AZ 85287 USA; 4 Arizona State University Health Services, Arizona State University, Tempe, AZ 85287 USA; 5 Biodesign Center for Fundamental and Applied Microbiomics, Center for Evolution and Medicine, School of Life Sciences, Arizona State University, Tempe, AZ 85287 USA; 6 College of Health Solutions, Arizona State University, Phoenix, AZ 85004 USA

**Keywords:** Influenza virus, *Orthomyxoviridae*, HTS, Variant analysis, Local transmission, Arizona

## Abstract

**Background:**

Local transmission of seasonal influenza viruses (IVs) can be difficult to resolve. Here, we study if coupling high-throughput sequencing (HTS) of hemagglutinin (HA) and neuraminidase (NA) genes with variant analysis can resolve strains from local transmission that have identical consensus genome. We analyzed 24 samples collected over four days in January 2020 at a large university in the US. We amplified complete hemagglutinin (HA) and neuraminidase (NA) genomic segments followed by Illumina sequencing. We identified consensus complete HA and NA segments using BLASTn and performed variant analysis on strains whose HA and NA segments were 100% similar.

**Results:**

Twelve of the 24 samples were PCR positive, and we detected complete HA and/or NA segments by de novo assembly in 83.33% (10/12) of them. Similarity and phylogenetic analysis showed that 70% (7/10) of the strains were distinct while the remaining 30% had identical consensus sequences. These three samples also had IAV and IBV co-infection. However, subsequent variant analysis showed that they had distinct variant profiles. While the IAV HA of one sample had no variant, another had a T663C mutation and another had both C1379T and C1589A.

**Conclusion:**

In this study, we showed that HTS coupled with variant analysis of only HA and NA genes can help resolve variants that are closely related. We also provide evidence that during a short time period in the 2019–2020 season, co-infection of IAV and IBV occurred on the university campus and both 2020/2021 and 2021/2022 WHO recommended H1N1 vaccine strains were co-circulating.

**Supplementary Information:**

The online version contains supplementary material available at 10.1186/s12879-021-06526-5.

## Introduction

Influenza viruses (IVs) are negative sense, segmented single-stranded RNA viruses in the family *Orthomyxoviridae* [7]. There are seven genera within the family and a total of nine species have been assigned to these genera [13]. The species *Influenza A virus* is assigned to the genus *Alphainfluenzavirus* whereas *Influenza B virus* to *Betainfluenzavirus*. Influenza A viruses (IAVs) and influenza B viruses (IBVs) cause acute respiratory illness in humans and animals and are responsible for yearly epidemics with significant morbidity and mortality [22]. Specifically, during the 2019–2020 IV season in the United States, it is estimated that IV was associated with 38 million illnesses, 18 million medical visits, 405,000 hospitalizations, and 22,000 deaths [9]. More importantly, IAVs have resulted in at least four pandemics [15, 30] and there is heightened anticipation that another might be looming [31].

Evolution of the influenza viruses (IVs) is a culmination of the effect of selective pressure on the variants generated by the combination of an error prone RNA dependent RNA polymerase and reassortment of genomic segments during co-infection. Influenza virus molecular clock estimations show that individual genomic segments of different species evolve at different rates, on the average 2.6 × 10^–3^ substitutions/site/year for IAV and 0.5 × 10^–3^ substitutions/site/year for IBV (i.e., about fivefold difference between the evolutionary rate of both viruses) [23]. This stipulates that if a chain of transmission is monitored, every 1000 nt of IAV or IBV genome is expected to acquire fixed mutations at a rate of about 2.6 or 0.5, per year and 5.2 or 1, per 2000 nt/year, respectively. Though the different genomic segments of the IV genome evolve at different rates, on average, this should be less than one substitution/month/segment. At this rate we may see genomes that belong to the same or related transmission chains remain unchanged at the consensus level over a period of 2–4 weeks. In fact, studies have documented the same IV genome recovered from cases sampled less than 10 days apart in the same community [12, 19, 20, 27]. Nonetheless, while the molecular clock (which determines the rate at which mutations become fixed or present within all individuals in a population) reflects the way genomes of IVs evolve at a population or global level, intra-host diversity of IVs is better captured by mutation rate (the rate at which errors are made during replication of the viral genome) [18, 25].

It has been suggested that the intra-host population of RNA viruses is actually a nonrandom population of variants and only a subset of this population eventually succeed in establishing an infection in a new host due to transmission bottlenecks [14]. The application of high throughput sequencing (HTS) now enables us to determine these variants [14, 20, 27]. Specifically, amplicon-based HTS of complete genomes of IVs [38, 40] has been recently applied to differentiate variants that have identical consensus genomes, examine their local transmission chains, and quantifying between host transmission [20, 27]. A problem with this approach, however, is the need for at least 10^3^ genomic equivalents for reliable and reproducible results [20, 33]. Zhou et al. [39] described a new assay that can detect 1–10 TCID_50_/RT-PCR but only targets coding segments (HA and NA [and M, if the primers are included]) essential for antigenic and antiviral surveillance rather than all eight segments. In this study, we address whether HTS of only the complete HA and NA coding segments can help differentiate variants of IVs that are closely related. We use amplicon-based HTS targeting the complete HA and NA coding segments on 24 IV positive nasal pharyngeal swabs collected over a four-day period on a university campus in Arizona, USA. From these we determined complete HA and NA segments using the Zhou et al. [39] approach and undertook both phylogenetic and variant analyses. We found that closely related IV strains (even those with the same consensus sequence) could be delineated using this approach for the HA and NA coding segments.

## Methods and materials

### Sample collection

Over the course of four (4) days (21st–24th of January 2020) we received twenty-four (24) influenza virus positive samples (6 samples each day for four days) from the on-campus clinic at Arizona State University (ASU) in Tempe (Arizona, USA) as part of an ongoing influenza virus surveillance study. The samples were determined influenza virus positive at the clinic using a rapid detection platform (Abbott ID Now, USA), anonymized and subsequently transferred to our laboratory while maintaining the cold-chain.

### RNA extraction and cDNA synthesis

Total RNA was extracted from the 24 samples using the viral RNA extraction kit (Qiagen, USA) in accordance with manufacturer’s instructions. The extracted RNA was used as template for cDNA synthesis using the SuperScript™ IV First-Strand Synthesis cDNA synthesis kit (Invitrogen, USA) following manufacturer’s instructions with a final reaction volume of 20 μl per sample. First, we mixed 5 μl of extracted RNA with 1 μl each of primers Uni12 [11] and B-HANA-F [39], 1 μl of dNTPs and 5 μl of nuclease-free water. We incubated the mixture at 75 °C for 5 min and subsequently cooled it on ice for one minute. Following this, we added 7 μl of a mixture containing RT-Buffer (4 μl), DTT (1 μl), RNase inhibitor (1 μl) and RT-SSIV enzyme (1 μl) to each vial. Using the BioRad C1000 thermal cycler (BioRad, USA), we performed reverse transcription with the following reaction conditions; 23 °C for 10 min, 55 °C for 45 min, 80 °C for 10 min and 4 °C for 5 min. We used the resultant cDNA for downstream analysis or stored it at −20 °C until needed.

### Complete HA and NA segment amplification PCR and Illumina sequencing

Using a modified Zhou et al. [39] protocol, we amplified the HA and NA coding segments from each of the 24 samples with the primers outlined in their protocol. For each sample, we undertook both influenza-A and influenza-B specific amplifications in 25 μl reaction volumes. The influenza-A assay was undertaken with 2 μl of cDNA, 12.5 μl of 2X RT-PCR buffer, 0.5 μl of the RT/Hifi enzyme, 8.5 μl of nuclease-free water, 3 pmol of each of the influenza-A HA forward and reverse primers, and 4.5 pmol of each of the influenza-A NA forward and reverse primers. The influenza-B assay was undertaken with 2 μl of cDNA, 12.5 μl of 2X RT-PCR buffer, 0.5 μl of the RT/Hifi enzyme, 8.8 μl of nuclease-free water, and 6 pmol of each of the influenza-B-HANA forward and reverse primers. The following thermal cycling conditions were used for the polymerase chain reaction (PCR) amplification using the BioRad C1000 thermal cycler (BioRad, USA); 94 °C for 2 min, 5 cycles of 94 °C for 30 s, 45 °C for 30 s and 68 °C for 3 min; 35 cycles of 94 °C for 30 s, 57 °C for 30 s and 68 °C for 3 min; followed by 68 °C for 10 min and 12 °C till stopped.

The amplicons were pooled by sample and 5 μl of each was resolved on a 1% agarose gel. For those with amplicons, a PCR cleanup was carried out using the Qiagen PCR cleanup kit (Qiagen, Germany) according to manufacturer’s instructions. Concentration of the amplicons for each sample was determined using the Nanodrop 2000 (Thermo Fisher Scientific, USA) and 15 μl of the cleaned PCR product per sample was processed by the ASU Genomics Core (Arizona, USA) for library preparation (KAPA Hyperplus Library Kit, Roche, USA) and paired-end sequencing (2 × 250 bp) on an Illumina MiSeq sequencer V2 (Illumina, USA). The IV sequences generated in this study have been deposited in GenBank with the following accession numbers MW286383, MW286387-MW286389, MW286401, MW288644, MW288645, MW288651, MW288656, MW288666, MW288667, MW288670-MW288672, MW288689, MW288690, MW534549-MW534551, MW555993-MW555999.

### De novo and scaffold-based assembly and variant analysis

The raw reads were checked for quality using FASTQC [4] and de novo assembled using both MEGAHIT v1.2.9 and metaSpades v3.13.0 (independently) on the KBase platform [5] with default parameters and a minimum contig length of 1000 nucleotides. The resulting contigs were analyzed using BLASTn [3] and the influenza virus annotation tool [6] for virus type and segment identification. Scaffold-based assembly was undertaken using the BBmap plugin [8] in Geneious Prime software (Biomatters Ltd, New Zealand) with default settings. Variant analysis was performed using the variant tool in Geneious Prime with default settings.

### Phylogenetic and similarity analyses

We used the Influenza Research Database (IRD) [37] and ZooPhy [29] to genetically characterize the HA and NA coding segment sequences from this study. We searched for HA and NA segments of seasonal IAVs and IBVs that were sampled in the United States between the 1st and 31st of January 2020 and with IRD we removed duplicate sequences. IAV and IBV sequences recovered from cases in Arizona between 1 and 31st of January 2020 (if not already captured in the previous search or lost to de-duplication) were also downloaded and no de-duplication was undertaken.

**Fig. 1:**
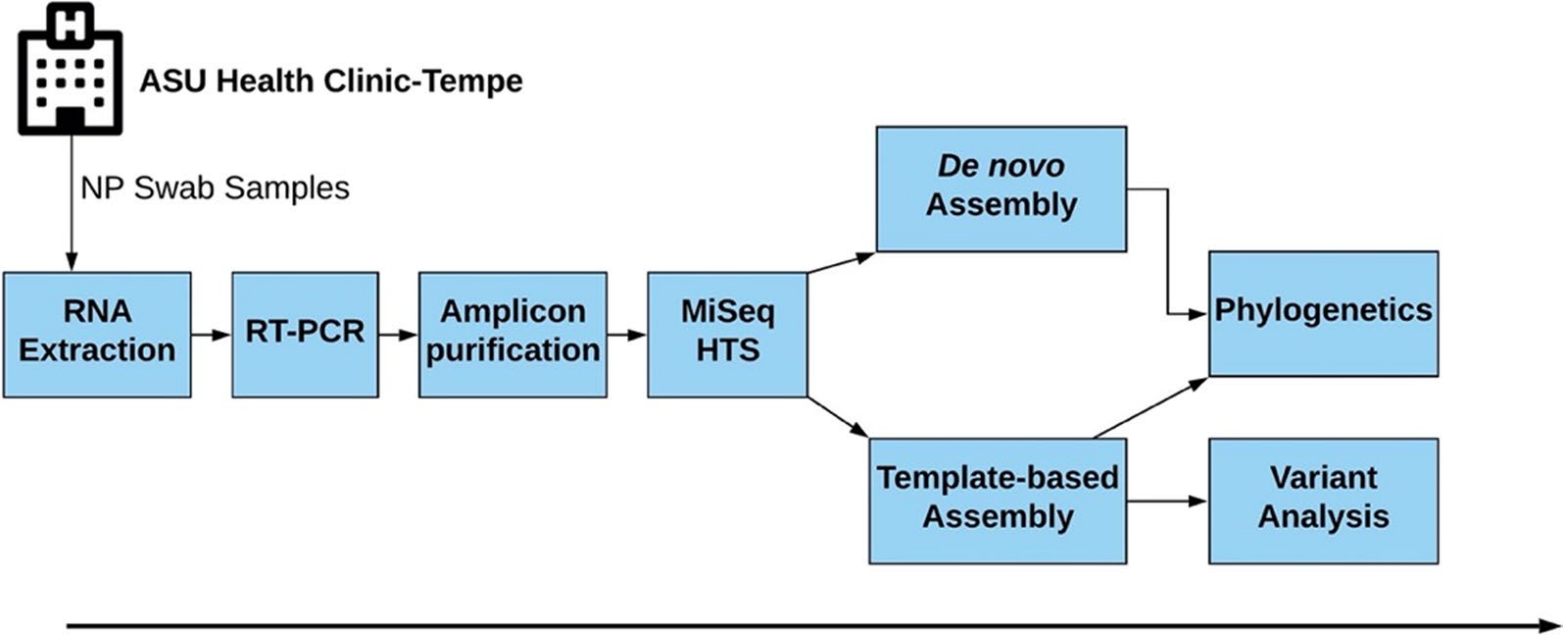
A schematic summary of the workflow followed in this study

We performed multiple sequence alignment of the entire dataset (as well as an independent run of just our new sequences) using the MUSCLE program in MEGA X software [16]. The alignment was used to infer a neighbor-joining (NJ) tree using the Tamura-Nei (TN) model with 1000 bootstrap replicates in MEGA X [16]. The alignment was also used to determine the pairwise identities using the Kimura 2-parameter model in MEGA X [16]. A schematic summary of the workflow followed in this study is depicted in Fig. 1.

## Results

Of the 24 samples subjected to the complete HA and NA segment PCR amplification, at least one of the expected band sizes representative of the targeted gene segments, HA or NA, was detected in 12 (50%) of the samples. Specifically, samples 2 and 3 (day 1, January 21); 7, 10 and 11 (day 2, January 22); 14, 16, 17 and 18 (day 3, January 23); 22, 23 and 24 (day 4, January 24) yielded at least one of the expected gene segments. Of these 12, we detected complete HA and/or NA segments by de novo assembly in 83.33% (10/12) (Table 1).

**Table 1:**
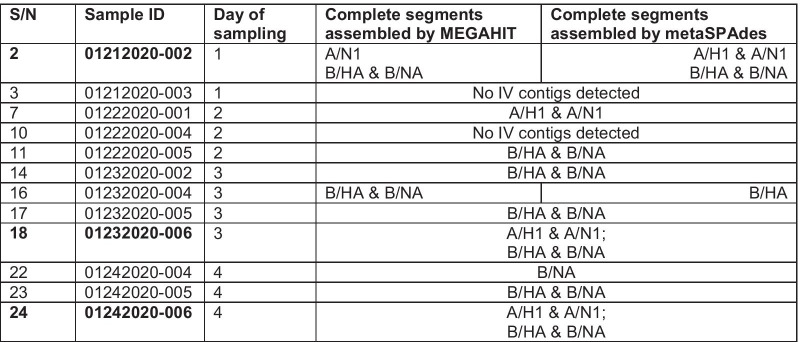
Complete IV HA and NA segments recovered by de novo assembly using MEGAHIT and metaSPAdes

We merged the cells to show agreements between the two assembly methods. We designate influenza B or the subtype for influenza A (as determined by BLASTn). Bold text for sample number and Sample ID indicates two virus types found

We also note some disagreements between the two assembly methods (Table 1) particularly in samples 2 and 16 (which could be consequent of the 1000nt contig cut off). For sample 2, three (3) segments (A/N1, B/HA and B/NA) were detected in contigs from MEGAHIT assembly, while all four segments (influenza A and B HA and NA) were detected in contigs from metaSpades assembly. For sample 16, B/HA and B/NA were detected in contigs from MEGAHIT assembly, while only B/HA was detected in contigs from metaSpades assembly.

In Additional file 1: Table S1, we show the results of our scaffold-based assembly using the IAV and IBV HA and NA contigs from Sample 2 (being the first sample in the set from which all four segments were recovered) (Additional file 1: Table S1). The results of scaffold-based assembly confirmed the results of de novo assembly. Sample 14 is peculiar in that despite the moderate number of reads for A/HA and A/NA we could not generate complete segments. Also, scaffold-based assembly showed that sample 22 had almost complete HA segment but with a 33nt gap at nucleotide position 1626 to 1658. This was therefore scaffolded to make the complete segment.

### Identification of IVs, phylogenetic analysis and alignment to vaccine strains

The IAV and IBV strains are summarized in Table 1. 90% (9/10) of the samples typed have IBV while 40% (4/10) have IAV. In Table 2 and Additional file 1: Table S2, we summarize the results of our BLASTn search which identified the four (4) IAVs as A(H1N1)pdm09 subtype and the nine IBVs as B/Victoria lineage viruses. In Tables 2 and 3 we show the three samples (2, 18, and 24) which contained both IAV and IBV HA and NA segments, have consensus segment sequences that are 100% identical and are 100% identical with the same sequence in GenBank. Furthermore, the consensus sequences from these three (3) samples cluster together on the respective phylogenetic trees (Additional file 1: Figure S1–S4).

**Table 2:** BLASTn results for our three samples that contained both influenza A and B sequences

S/N	Lab-ID	HA			NA		
		Most Similar Strain in GenBank (Accession No./date-of-collection)	Similarity (%)	Lineage	Most Similar Strain in GenBank (Accession No./date-of-collection)	Similarity (%)	Lineage
2	01212020–002	A/Washington/10/2020 (MT168520/01012020)	100	A (H1N1)pdm09	A/Washington/10/2020 (MT168522/01012020)	100	A(H1N1)pdm09
		B/Arizona/26/2019 (MN949149/11022019)	100	B/Victoria	B/Kenya/11/2019 (MN086301/04162019)	99.61	B/Victoria
18	01232020–006	A/Washington/10/2020 (MT168520/01012020)	100	A (H1N1)pdm09	A/Washington/10/2020 (MT168522/01012020)	100	A(H1N1)pdm09
		B/Arizona/26/2019 (MN949149/11022019)	100	B/Victoria	B/Kenya/11/2019 (MN086301/04162019)	99.61	B/Victoria
24	01242020–006	A/Washington/10/2020 (MT168520/01012020)	100	A (H1N1)pdm09	A/Washington/10/2020 (MT168522/01012020)	100	A(H1N1)pdm09
		B/Arizona/26/2019 (MN949149/11022019)	100	B/Victoria	B/Kenya/11/2019 (MN086301/04162019)	99.61	B/Victoria

**Table 3:** Pairwise similarity analysis showing only samples that are 100% similar to samples 2, 18, and 24. We bold comparisons that include samples beyond these three

Subtype	Sample numbers
A/H1	2 v. 18
	2 v. 24
	18 v. 24
A/N1	2 v. 18
	2 v. 24
	18 v. 24
B/HA	**2 v. 14**
	2 v. 18
	2 v. 24
	**14 v. 18**
	**14 v. 24**
	18 v. 24
B/NA	2 v. 18
	2 v. 24
	18 v. 24

**Fig. 2:**
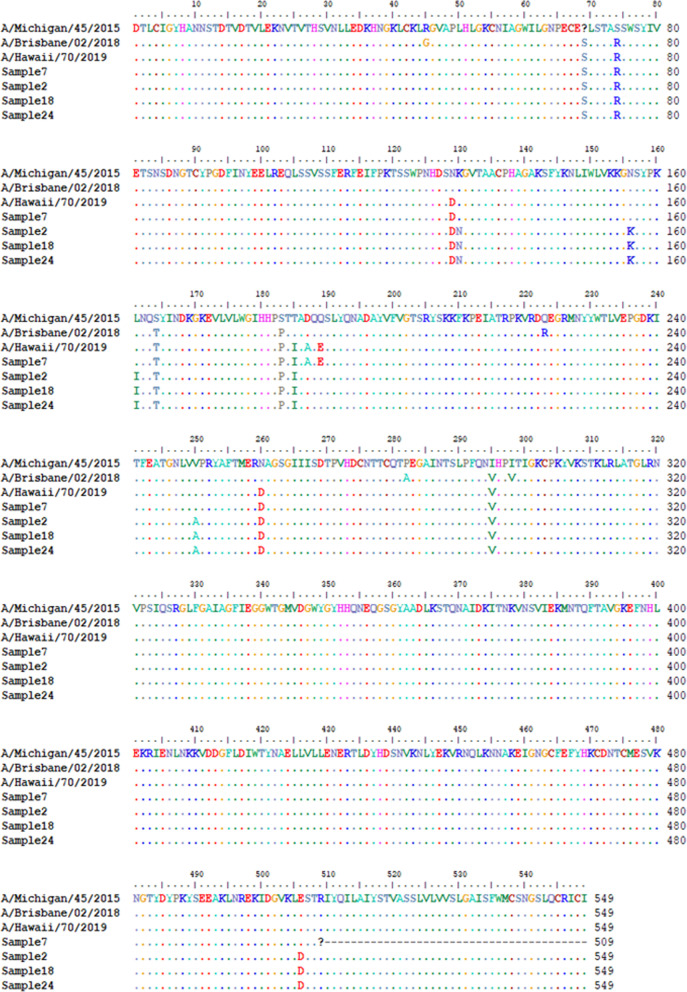
Alignment of IAV-HA samples 2, 7, 18 and 24 against the last two IAV H1N1 vaccine strains (A/Michigan/45/2015 and A/Brisbane/ 02/2018) and the current one (A/Hawaii/70/2019). Dots denote conservation while substitutions are highlighted by showing the new amino acid. Dashes denote missing sequence while question mark (?) suggest an ambiguous base was present in this codon hence hampering translation

Alignment of amino acid sequences of the IAV HA of the four (4) variants sequenced in this study with 2020/2021 reference vaccine strains [35] show that they belong to the phylogenetic clade 6B.1A, subclade 5A [35]. All four (4) variants have the HA amino acid substitutions N129D, S183P, T185I and N260D (Fig. 2) but belong to two lineages (5A-187A and 5A-156 K) [35, 36]. Sample 7 has two additional amino acid substitutions D187A and Q189E (Fig. 2 which show it belongs to the 5A-187A lineage and make it identical to the 2020/2021 WHO recommended northern hemisphere IAV H1N1 strains (A/Guangdong-Maonan/SWL1536/2019 and A/Hawaii/70/2019. Samples 2, 18 and 24 (which all have consensus sequences that are 100% identical lack these two substitutions, but have other substitutions; K130N, N156K, L161I, V250A and E506D that show they belong to the 5A-156 K lineage [36] and are antigenically identical to the 2021/2022 WHO recommended vaccine IAV H1N1 strains) (Fig. 2).

**Fig. 3:**
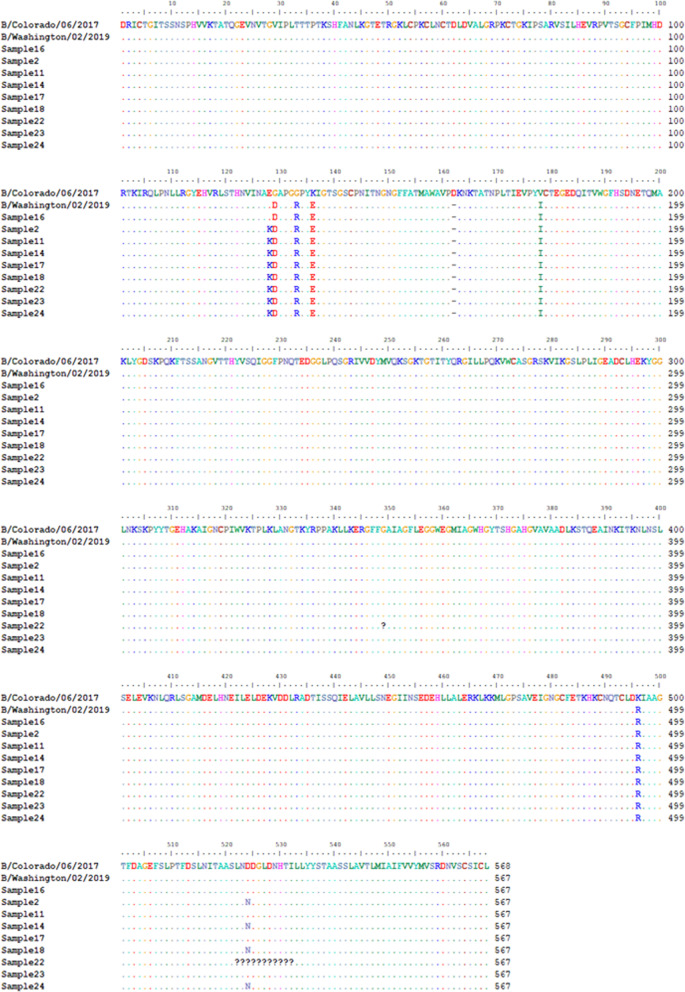
Alignment of IBV-HA samples 2, 11, 14, 16, 17, 18, 22, 23 and 24 against the last IBV vaccine strain (B/Colorado/06/2017) and the current one (B/Washington/02/2019). Dots denote conservation while substitutions are highlighted by showing the new amino acid. Dashes denote deletion. A single question mark (?) suggest an ambiguous base was present in this codon hence hampering translation. Multiple question marks denote a scaffold

Alignment of amino acid sequences of the IBV HA of the nine (9) variants recovered in this study with reference B/Victoria vaccine strains [35], show that they belong to the phylogenetic clade 1A just like B/Washington/02/2019 (which is the current [2020/2021] recommended vaccine IBV Victoria strain) [35]. In fact, sample 16 had the same substitutions as B/Washington/02/2019. All the remaining eight (8) variants however had an additional amino acid substitutions E128K (Fig. 3). In addition, four of the remaining eight had D524N (Fig. 3).

### Variant analysis

**Fig. 4:**
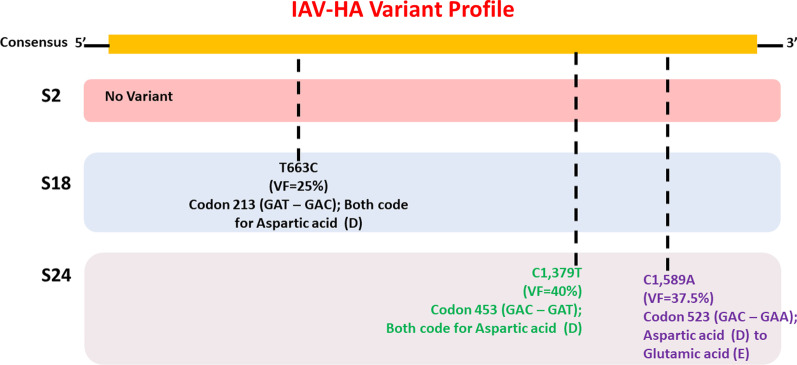
Variant profile of IAV-HA samples 2, 18 and 24. While no variant was found in sample #2, samples #18 and #24 have variants. Note that codons 213, 453 and 523 here are according to H1 numbering from first methionine. They would be codons 196, 436 and 506 respectively in Fig. 2 where H1 was numbered not from first methionine but without signal peptide

Analysis of the IAV-HA assemblies for samples 2, 18 and 24 showed that they were indeed different (Fig. 4). While sample 2 had no variants, samples 18 and 24 have variants distinguishing both from each other and from sample 2. Using default parameters (maximum variant p-value 10^–6^; i.e. 0.0001%), sample 24 had two variant positions identified; C1379T and C1589A (Fig. 4). Specifically, C1379T is a silent mutation (a transition) present at a frequency of 40%. It results in a change in codon 453 from GAC to GAT (silent mutation) with no consequent change in the amino acid encoded as both codons code for Aspartic acid (D). C1589A on the other hand is a missense mutation that results in a change in codon 523 from GAC to GAA and a consequent change in the amino acid encoded from aspartic acid (D) to glutamic acid (E). The C1589A variant position has a frequency of 37.5% (Fig. 4).

No variant was found in sample 18 using default parameters. However, when the maximum variant p-value was reduced to 10^–4^ (i.e., 0.01%), one variant (T663C) was detected. T663C is a silent mutation present at a frequency of 25%. It results in a change in codon 213 from GAT to GAC (silent mutation) with no consequent change in the amino acid encoded as both codons code for aspartic acid (D) (Fig. 4). Analyzing sample 24 with this reduced stringency resulted in two additional variants (G132A and a two-nucleotide deletion GT269-270) (data not shown).

No variants were found in IAV-NA, IBV HA and IBV NA even when the less stringent parameter was used. Hence, the only difference found in samples 2, 18 and 14 was in the variant profiles based on IAV-HA (Fig. 4).

### Neuraminidase sensitivity

All the neuraminidase sequences generated in this study (both IAV and IBV) lack amino acid changes associated with resistance to oseltamivir, peramivir and zanamivir [28, 34]. Q136 and H275 are conserved in all IAV-NA sequences determined in this study. Q136K has been associated with peramivir and zanamivir resistance while H275Y has been associated with resistance to oseltamivir and peramivir. Both substitutions (Q136K and H275Y) are absent in the IAV-NA variants described here (Additional file 1: Figure S5). In all IBV-NAs described here, H273 and R374 are conserved. H273Y has been associated with resistance to peramivir while R374K has been associated with resistance to oseltamivir, zanamivir and peramivir. Both substitutions (H273Y and R374K) are absent in the IBV-NA variants described in this study (Additional file 1: Figure S6).

## Discussion

### Sample resolution

In this study, we demonstrate that amplicon-based HTS of HA and NA segments (~ 24% [3300/ 13,800] of IV genomes) only (via Zhou et al., [39] protocol) can help resolve IV variants that are closely related (Fig. 4). It is important to note that the assay used here [39] which amplifies only HA and NA (~ 24% of IV genomes) is usually not routinely used for IV transmission studies where variant resolution is essential. Rather, complete genome amplicon-based HTS assays [38, 40] are used [20, 27]. While the focus of this study is not to compare the Zhou et al., [39] protocol with the complete genome amplicon-based HTS assays, when we subjected the 10 samples for which complete HA and NA contigs were recovered in this study (Table 1) to the complete genome assays, only sample #17 yielded bands of the expected size to the IBV complete genome assay (Additional file 1: Figure S8). This suggests that only sample #17 had virus concentration high enough to be positive for the IBV complete genome assay. Hence, if we used the complete genome assays, we would have recovered amplicons from only one of the ten samples described here.

It is crucial to emphasize, as previously mentioned in Zhou et al., [39], that the approach we use in this study for resolving variants with genome segments that are 100% identical also has immense potential for quick identification of IV strains responsible for local outbreaks and even novel IV strains with pandemic potential (such as pdm09 2009) because it enables the detection of both IAV and IBV, is less time consuming, cheaper (as more samples can be multiplexed), can produce more depth of coverage/sample, is more sensitive than the complete genome assays (hence, can assay low viral load samples) and is sufficient for antigenic and antiviral surveillance. Furthermore, we have described here an approach that allows decoupling of the single-tube version assay [39] such that choices can be made regarding the need to run whichever (IAV, IBV or both) component of the assay. This can be useful when prescreening has yielded insight in regards which IV type(s) is/are present in the sample.

Our findings confirm that both IAV and IBV strains were co-circulating in January 2020 (during the 2019/2020 influenza season) in Tempe (Arizona, USA; Table 2 and Additional file 1: Table S2). The A(H1N1) and IBV strains detected in this study were most closely related to A(H1N1)pdm09 and IBV Victoria lineage strains detected in the United States during the 2019/2020 influenza virus season (Table 2 and Additional file 1: Table S2). More importantly, they belong to the lineages (Figs. 2 and 3) documented to circulate globally during the 2019/2020 influenza virus season. In fact, the amino acid sequence of sample 7 IAV-HA and sample 16 IBV-HA (Figs. 2 and 3) show they might be antigenically identical to the vaccine strains selected by the WHO for the 2020/2021 influenza virus season [35]. Furthermore, the amino acid sequence of sample 2, 18 and 24 IAV-HA show they might be antigenically identical to the IAV-vaccine strains selected by the WHO for the 2021/2022 influenza virus season [36]. This shows that the samples analyzed in this study were part of the 2019/2020 influenza virus season, circulated into the 2020/2021 season and further contribute to the pool of sequenced strains available.

We rarely found that each sample’s HA and NA consensus sequences were in agreement on the most similar strain in GenBank (via a BLAST search). For example, the consensus HA sequence of sample 17 is most similar (99.81%) to that of A/Louisiana/09/2020 but its consensus NA sequence is most similar (99.86%) to that of A/New Hampshire/32/2020. This difference in similarity however, can be accounted for by four nucleotide changes. While sample 17 NA shares 1431/1433 identities with the NA of A/New Hampshire/32/2020, it shares 1427/1433 identities with the NA of A/Louisiana/09/2020. Considering the molecular clock of IVs [23], this difference at the consensus level cannot be acquired over the course of one infectious cycle. Rather, this pattern most likely suggests there must have been re-assortment between two IAV strains at some point to generate this strain. This dichotomy is even more pronounced in the IBV strains detected in this study (Table 2 and Additional file 1: Table S2) where while all the IBV-NAs described in this study seem to be most similar to B/Kenya/11/2019s NA (to varying degrees), none of the IBV-HAs has B/Kenya/11/2019s HA as the most similar sequence in GenBank. Rather, the IBV-HAs recovered here appear to be quite diverse. Our results therefore suggest that, local co-infection with multiple strains of IV might be very common [10, 27].

It is, however, difficult to track co-infection with similar or even identical strains, and this might be partly attributable to the fact that it is challenging to delineate co-infection by multiple strains of IV genotypes without any form of ‘barcoding’, as has been done in some experimental studies [1, 17, 21, 26]. This necessitates the need for unique sample variant profiling [20, 27] as a form of ‘barcoding’.

### Unique sample variant profiles as ‘barcodes’

Using variant profiling we were able to show in this study, that samples 2, 18 and 24 were different samples (based on IAV-HA) (Fig. 4). In fact, though the IBV-NAs of samples 11 and 23 already show them to be different variants (Additional file 1: Table S2), we were also able to resolve their HAs which were 100% identical at the consensus level (Additional file 1: Figure S7). Specifically, both differ by a variant T568C which is a silent mutation but is only present in sample 23 and at a frequency of 42% (Additional file 1: Figure S7). Hence, the findings of this study provide proof that IV samples collected over a short period of time (four days in this case), that likely belong to the same transmission chain and/or have the same consensus sequence can be delineated as independent isolates using variant profiling of only HA and NA as a form of ‘barcoding’.

We note that not all of the variants discovered here are biologically meaningful. A good example is the di-nucleotide deletion (GT269-270) in sample 24. While this might likely result in a frame shift, it remains part of the signature specific to sample 24. This exemplifies the need to think about sample specific variant signatures beyond biologically meaningful mutations which ordinarily will be selected against and consequently unlikely to be propagated to subsequent generations. It is expected that several variants will be produced during replication that might carry beneficial, silent or deleterious mutations [25]. Variant analysis might capture some or all of this when present. Hence, such should not be considered aberrations when present and captured as part of the samples variant profile.

### Co-infection and possible co-transmission

We identified at least seven different transmission chains (Table 2 and Additional file 1: Table S2) from our samples that were collected over four days. In one of the possible transmission clusters identified (Table 2 and Additional file 1: Figures S1–S4), each sample had IAV and IBV that were not distinguishable based on consensus sequences of their HA and NA. These three samples were collected over the course of four days. Sample 2 and 18 were collected 2-days apart, while samples 2 and 24 were collected 3 days apart. We note the limitation that we cannot categorically state whether these three samples were linked (as our study samples were anonymized). However, the serial interval (time from symptom onset in index case to symptom onset in secondary case) of 2–3 days [32] coupled with the completely indistinguishable consensus sequence data is consistent with patterns found in household or close contact transmission of IV [12, 19, 20, 27]. While studies have documented IAV and IBV co-infection [2, 24], few have suggested the possibility of co-transmission of different strains [19, 27]. We understand that the data provided by samples 2, 18 and 24 may be the consequence of independent but sequential infection by IAV and IBV, it is equally likely that co-transmission of IAV and IBV (two different species) might have happened during the sampling period and we most likely documented some nodes in the transmission chain (Table 3 and Fig. 2, S1-S4).

## Conclusion

In this study, we set out to determine whether the Zhou et al. [39] amplicon-based HTS of HA and NA coding segments only can help resolve variants of IV that are closely related. Specifically, variants that have the same consensus sequence. Our findings confirm that this is possible. However, considering we have only resolved a few samples with identical consensus sequence in this study, further work is needed using a larger number of samples with identical consensus sequences. Our findings also provide anecdotal evidence that co-transmission of IAV and IBV might have happened during the period sampled and we might have documented some nodes in the transmission chain. Finally, we show that during the short time period sampled in this study both 2020/2021 and 2021/2022 WHO recommended H1N1 vaccine strains were co-circulating in Tempe, Arizona.

## Supplementary Information

**Additional file 1: Table S1.** Number of mapped reads mapped using BBmap per influenza A and influenza B HA and NA segments per sample. Reads which did not result in a complete segment are shown in bold font. Please note that no mean coverage data is included in this table for samples from which complete segments could not be assembled. **Table S2.** BLASTn result for the samples in this study in which a single influenza type was detected (and not included in Table 2). **Figure S1.** Phylogenetic tree of genetic relationship between IAV H1N1 (pdm09) HA contigs detected in this study and those detected in USA between January1st and 31st 2020. The cluster of the three variants (samples 2, 18 and 24) with 100% identity in consensus sequence (Table 3) is highlighted. The three (3) variants are indicated with black triangle and bootstrap values are indicated if > 50%. **Figure S2.** Phylogenetic tree of genetic relationship between IAV H1N1 (pdm09) NA contigs detected in this study and those detected in USA between January1st and 31st 2020. The cluster of the three variants (samples 2, 18 and 24) with 100% identity in consensus sequence (Table 3) is highlighted. The three (3) variants are indicated with black triangle and bootstrap values are indicated if > 50%. **Figure S3.** Phylogenetic tree of genetic relationship between IBV HA contigs detected in this study and those detected in USA between January 1st and 31st 2020. The cluster of the three variants (samples 2, 18 and 24) with 100% identity in consensus sequence (Table 3 and Additional file 1: Table S2) is highlighted. Note that a four variant (sample 14) also belongs to this cluster but only its’ HA. Its’ NA is however different from those of Samples 2, 18 and 24 (Additional file 1: Table S2). The four (4) variants are indicated with black triangle and bootstrap values are indicated if > 50%. **Figure S4.** Phylogenetic tree of genetic relationship between IBV NA contigs detected in this study and those detected in USA between January1st and 31st 2020. The cluster of the three variants (samples 2, 18 and 24) with 100% identity in consensus sequence (Table 3 and Additional file 1: Table S2) is highlighted. Note that a 4th variant (sample 14) also belongs to this cluster but only its’ HA. Its’ NA is however different from those of Samples 2, 18 and 24 (Additional file 1: Table S2). The three (3) variants (samples 2, 18 and 24) are indicated with black triangle and bootstrap values are indicated if > 50%. **Figure S5.** Alignment of Neuraminidase of IAV. Note Q136 and H275 which are conserved in all IAV-NA sequences generated in this study. Q136K has been associated with Peramivir and Zanamivir resistance while H275Y has been associated with resistance to Oseltamivir and Peramivir. Both substitutions are absent in the variants described in this study (https://www.who.int/influenza/gisrs_laboratory/antiviral_susceptibility/NAI_Reduced_Susceptibility_Marker_Table_WHO.pdf?ua=1). **Figure S6**. Alignment of Neuraminidase of IBV. Note H273 and R374 which are conserved in all IBV-NA sequences generated in this study. H273Y has been associated with resistance to Peramivir while R374K has been associated with resistance to Oseltamivir, Zanamivir and Peramivir. Both substitutions are absent in the variants described in this study (https://www.who.int/influenza/gisrs_laboratory/antiviral_susceptibility/NAI_Reduced_Susceptibility_Marker_Table_WHO.pdf?ua=1). **Figure S7.** Variant profile of IBV-HA samples 11 and 23. Samples 11 and 23 share two variants C121T and T1018C. A third variant (T568C) distinguishes them from each other. Note that codons 34, 183 and 333 here are according to H1 numbering from first methionine. They would be codon 19, 169 and 319 in Fig. 3 where H1 is numbered not from first methionine but without signal peptide. **Figure S8**. Gel electrophoresis result of the FluB complete genome assay [39]. All 10 samples from which complete HA and NA segments were recovered in this study (Table 1) were subjected to this assay. Lanes 1 and 13 contain molecular ladder. Lane 12 has negative control. Lanes 14 and 15 show the expected band patterns for IAV and IBV complete genome positive samples. Note that lane 15 has the positive control for this assay. Lane 14 has positive control for the IAV complete genome assay. It was loaded onto this gel only for comparison of the band patterns. Note sample 17 (lane 7) is the only sample positive for the assay. Please see [38] and [40] for detailed protocols for the IAV and IBV complete genome amplification assays.

## Data Availability

Sequences produced from this study have been deposited in the NIH GenBank databases under the following accession numbers: MW286383, MW286387-MW286389, MW286401, MW288644, MW288645, MW288651, MW288656, MW288666, MW288667, MW288670-MW288672, MW288689, MW288690, MW534549-MW534551, MW555993-MW555999. https://www.ncbi.nlm.nih.gov/nuccore/MW286383,MW286387,MW286389,MW286401,MW288644,MW288645,MW288651,MW288656,MW288666,MW288667,MW288670,MW288671,MW288672,MW288689,MW288690,MW534549,MW534550,MW534551,MW555993,MW555994,MW555995,MW555996,MW555997,MW555998,MW555999
