# Supplementary material for: Use of hemagglutinin and neuraminidase amplicon-based high-throughput sequencing with variant analysis to detect co-infection and resolve identical consensus sequences of seasonal influenza in a university setting
Source: BMC Infect Dis. 2021 Aug 13;21:810. doi: 10.1186/s12879-021-06526-5 (PMC8360813; doi:10.1186/s12879-021-06526-5)
Supplement: Supplementary file 1 — Additional file 1: Table S1. Number of mapped reads mapped using BBmap per influenza A and influenza B HA and NA segments per sample. Reads which did not result in a complete segment are shown in bold font. Please note that no mean coverage data is included in this table for samples from which complete segments could not be assembled. Table S2. BLASTn result for the samples in this study in which a single influenza type was detected (and not included in Table 2). Figure S1. Phylogenetic tree of genetic relationship between IAV H1N1 (pdm09) HA contigs detected in this study and those detected in USA between January1st and 31st 2020. The cluster of the three variants (samples 2, 18 and 24) with 100% identity in consensus sequence (Table 3) is highlighted. The three (3) variants are indicated with black triangle and bootstrap values are indicated if > 50%. Figure S2. Phylogenetic tree of genetic relationship between IAV H1N1 (pdm09) NA contigs detected in this study and those detected in USA between January1st and 31st 2020. The cluster of the three variants (samples 2, 18 and 24) with 100% identity in consensus sequence (Table 3) is highlighted. The three (3) variants are indicated with black triangle and bootstrap values are indicated if > 50%. Figure S3. Phylogenetic tree of genetic relationship between IBV HA contigs detected in this study and those detected in USA between January 1st and 31st 2020. The cluster of the three variants (samples 2, 18 and 24) with 100% identity in consensus sequence (Table 3 and Additional file 1: Table S2) is highlighted. Note that a four variant (sample 14) also belongs to this cluster but only its’ HA. Its’ NA is however different from those of Samples 2, 18 and 24 (Additional file 1: Table S2). The four (4) variants are indicated with black triangle and bootstrap values are indicated if > 50%. Figure S4. Phylogenetic tree of genetic relationship between IBV NA contigs detected in this study and those detected in USA between January [file 12879_2021_6526_MOESM1_ESM.docx]

**Supplemental Tables and Figures**

**Table S1. Number of mapped reads mapped using BBmap per influenza A and influenza B HA and NA segments per sample.** Reads which did not result in a complete segment are shown in bold font. Please note that no mean coverage data is included in this table for samples from which complete segments could not be assembled.

| **S/N** | **Lab-ID** | **Influenza A**  **(mean coverage)** | | **Influenza B**  **(mean coverage)** | | **Total # of reads** |  |
| --- | --- | --- | --- | --- | --- | --- | --- |
|  |  | **HA** | **NA** | **HA** | **NA** |  |  |
| 2 | 01212020-002 | 78,643 **(~22,444X)** | 134,661 **(~46,985X)** | 1,281 **(~347X)** | 1,439 **(~462X)** | 1,370,974 |  |
| 7 | 01222020-001 | 225 **(~64X)** | 195 **(~68X)** | **7** | **7** | 1,080,624 |  |
| 11 | 01222020-005 | 0 | 0 | 308 **(~83X)** | 309 **(~99X)** | 1,216,564 |  |
| 14 | 01232020-002 | **2,123** | **1,259** | 79 **(~21X)** | 96 **(~31X)** | 1,196,916 |  |
| 16 | 01232020-004 | 0 | **2** | 2,153 **(~583X)** | 19,531 **(~6,272X)** | 1,338,544 |  |
| 17 | 01232020-005 | 0 | **1** | 660,765 **(~178,875X)** | 687,941 **(~220,919X)** | 1,670,554 |  |
| 18 | 01232020-006 | 155 **(~44X)** | 301 **(~105X)** | 1,127 **(~305X)** | 1,364 **(~438X)** | 1,345,692 |  |
| 22* | 01242020-004 | 0 | **3** | 101 **(~27X)** | 382 **(~123X)** | 1,558,558 |  |
| 23 | 01242020-005 | 0 | **854** | 168 **(~45X)** | 196 **(~63X)** | 1,372,020 |  |
| 24 | 01242020-006 | 101 **(~29X)** | 181 **(~63X)** | 1,380 **(~374X)** | 1,719 **(~552X)** | 1,299,914 |  |
| *33nt missing | | | | | | | |

**Table S2. BLASTn result for the samples in this study in which a single influenza type was detected (and not included in Table 2).**

| **S/N** | **Lab-ID** | **HA** | | | **NA** | | |
| --- | --- | --- | --- | --- | --- | --- | --- |
|  |  | **Most Similar Strain in GenBank** | **Similarity (%)** | **Lineage** | **Most Similar Strain in GenBank** | **Similarity (%)** | **Lineage** |
| 7 | 01222020-001 | A/Louisiana/09/2020  (MT303464) | 99.81 | A(H1N1)pdm09 | A/New Hampshire/32/2019  (MN947997) | 99.86 | A(H1N1)pdm09 |
| 16 | 01232020-004 | B/Washington/02/2019  (MK676294) | 100 | B/Victoria | B/Kenya/11/2019  (MN086301) | 99.74 | B/Victoria |
| 22 | 01242020-004 | B/Michigan/101/2020  (MT581864) | 99.82 | B/Victoria | B/Kenya/11/2019  (MN086301) | 99.68 | B/Victoria |
| 11 | 01222020-005 | B/Iowa/36/2019  (MT314977) | 99.95 | B/Victoria | B/Kenya/11/2019  (MN086301) | 99.68 | B/Victoria |
| 23 | 01242020-005 | B/Iowa/36/2019  (MT314977) | 99.95 | B/Victoria | B/Kenya/11/2019  (MN086301) | 99.61 | B/Victoria |
| 14 | 01232020-002 | B/Arizona/26/2019  (MN949149) | 100 | B/Victoria | B/Kenya/11/2019  (MN086301) | 99.55 | B/Victoria |
| 17 | 01232020-005 | B/Arizona/33/2019  (MT029446) | 99.95 | B/Victoria | B/Kenya/11/2019  (MN086301) | 99.61 | B/Victoria |


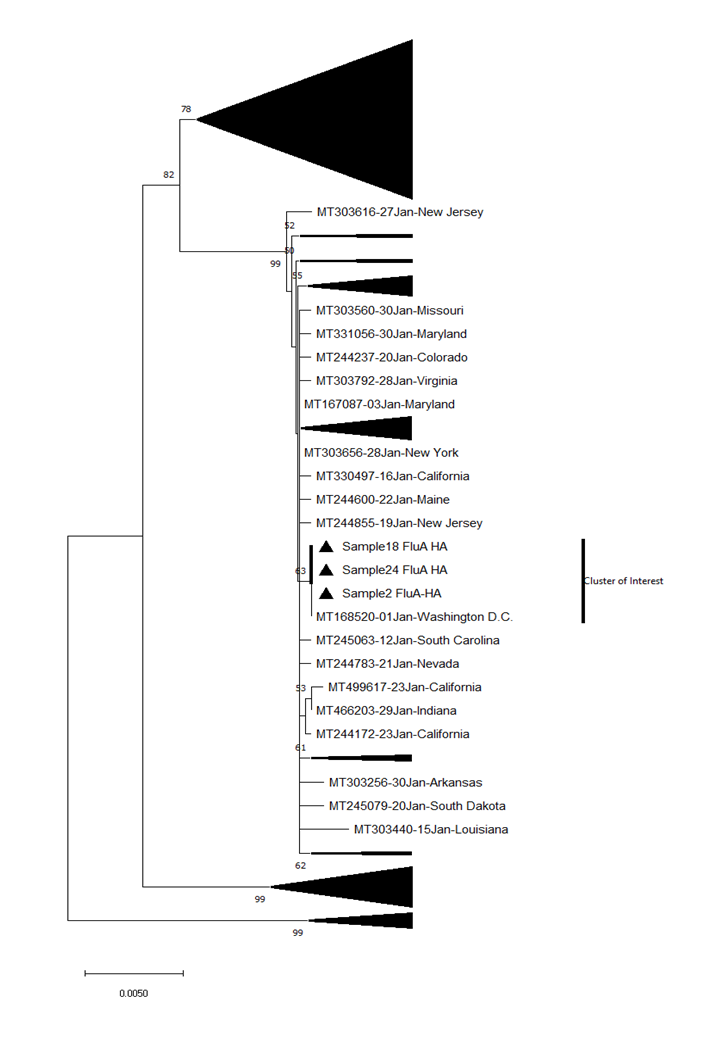


**Figure S1:** **Phylogenetic tree of genetic relationship between IAV H1N1 (pdm09) HA contigs detected in this study and those detected in USA between January1st and 31^st^ 2020.** The cluster of the three variants (samples 2, 18 and 24) with 100% identity in consensus sequence (Table 3) is highlighted. The three (3) variants are indicated with black triangle and bootstrap values are indicated if >50%.


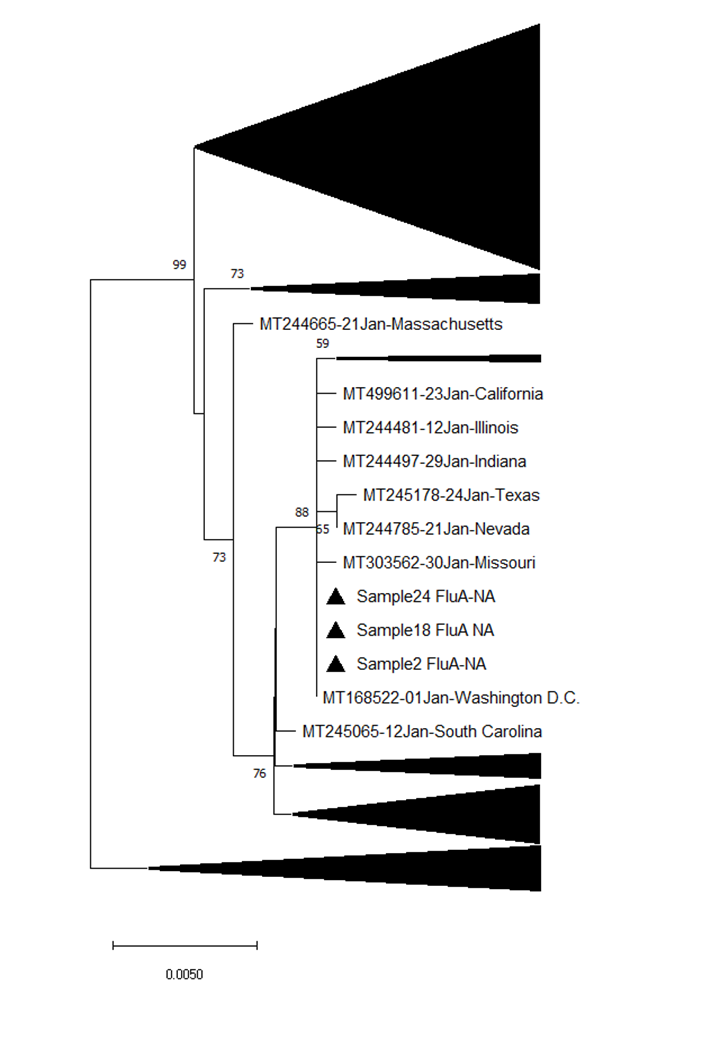


**Figure S2:** **Phylogenetic tree of genetic relationship between IAV H1N1 (pdm09) NA contigs detected in this study and those detected in USA between January1st and 31^st^ 2020.** The cluster of the three variants (samples 2, 18 and 24) with 100% identity in consensus sequence (Table 3) is highlighted. The three (3) variants are indicated with black triangle and bootstrap values are indicated if >50%.


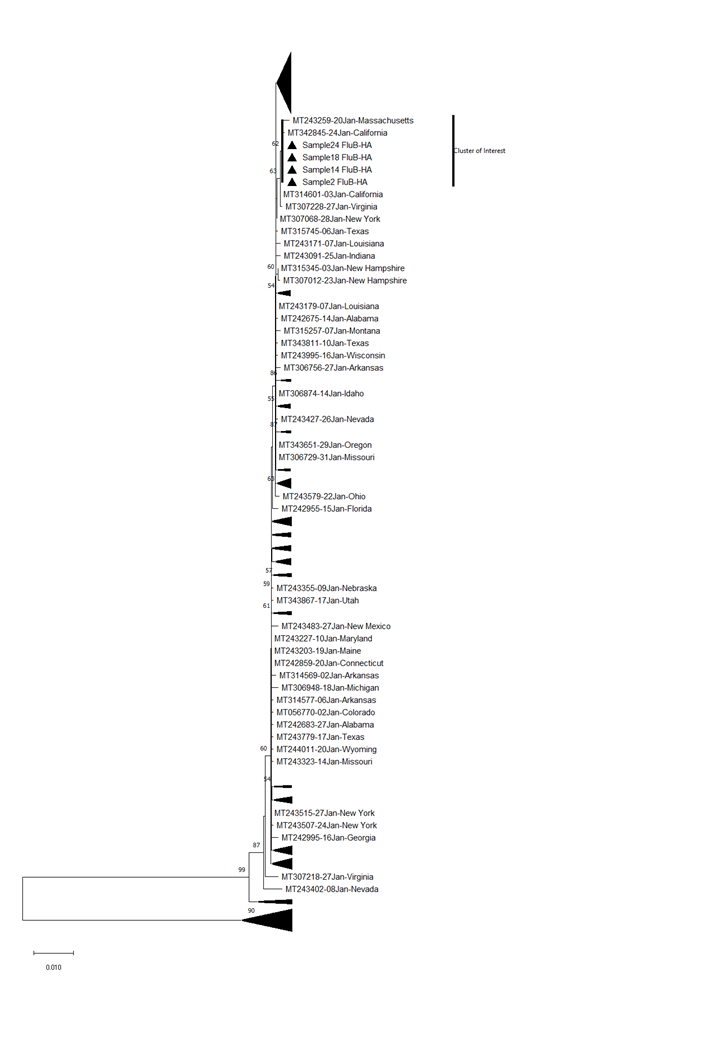


**Figure S3:** **Phylogenetic tree of genetic relationship between IBV HA contigs detected in this study and those detected in USA between January1st and 31^st^ 2020.** The cluster of the three variants (samples 2, 18 and 24) with 100% identity in consensus sequence (Tables 3 and S2) is highlighted. Note that a four variant (sample 14) also belongs to this cluster but only its’ HA. Its’ NA is however different from those of Samples 2, 18 and 24 (Table S2). The four (4) variants are indicated with black triangle and bootstrap values are indicated if >50%.


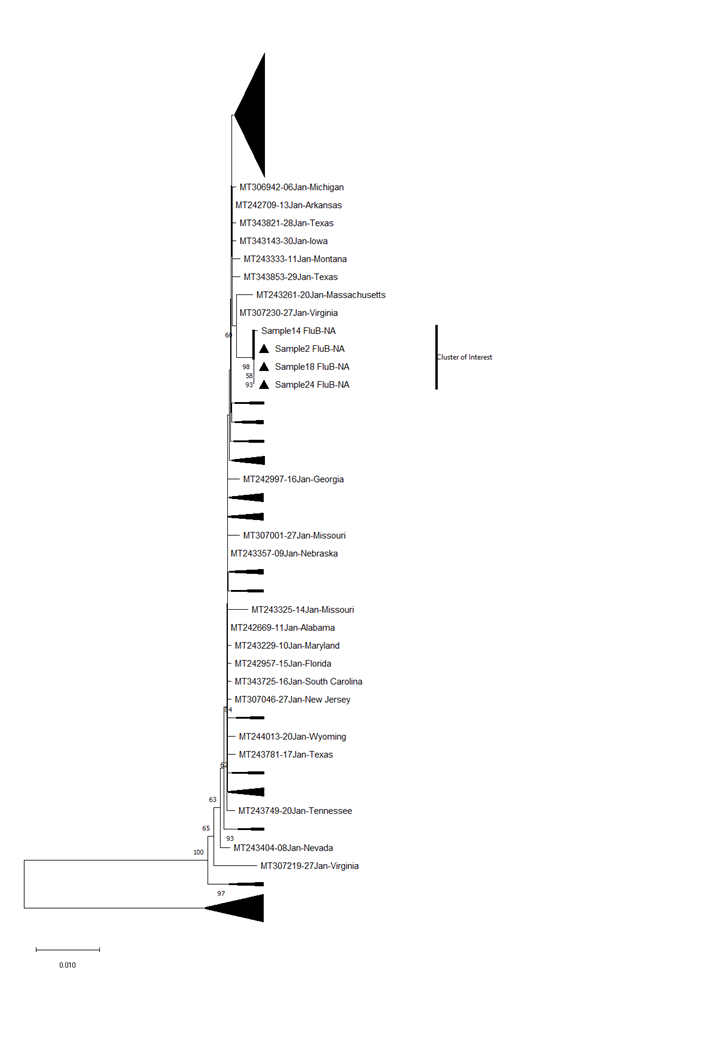


**Figure S4:** **Phylogenetic tree of genetic relationship between IBV NA contigs detected in this study and those detected in USA between January1st and 31^st^ 2020.** The cluster of the three variants (samples 2, 18 and 24) with 100% identity in consensus sequence (Tables 3 and S2) is highlighted. Note that a 4th variant (sample 14) also belongs to this cluster but only its’ HA. Its’ NA is however different from those of Samples 2, 18 and 24 (Table S2). The three (3) variants (samples 2, 18 and 24) are indicated with black triangle and bootstrap values are indicated if >50%.


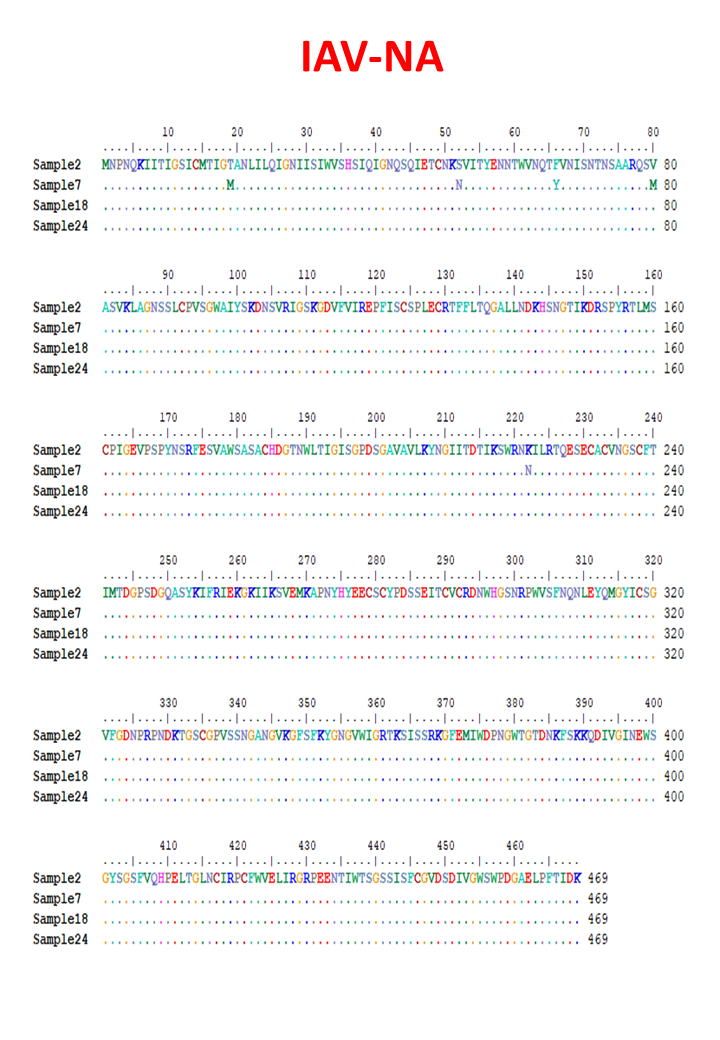


**Figure S5:** **Alignment of Neuraminidase of IAV .** Note Q136 and H275 which are conserved in all IAV-NA sequences generated in this study. Q136K has been associated with Peramivir and Zanamivir resistance while H275Y has been associated with resistance to Oseltamivir and Peramivir. Both substitutions are absent in the variants described in this study (<https://www.who.int/influenza/gisrs_laboratory/antiviral_susceptibility/NAI_Reduced_Susceptibility_Marker_Table_WHO.pdf?ua=1>).


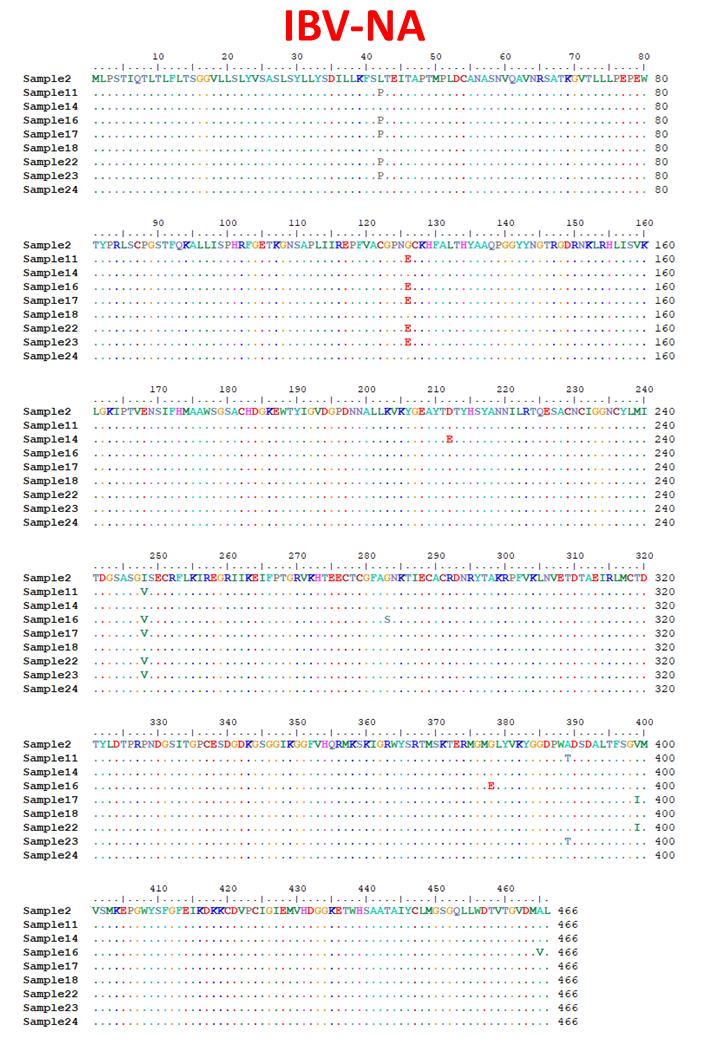


**Figure S6:** **Alignment of Neuraminidase of IBV.** Note H273 and R374 which are conserved in all IBV-NA sequences generated in this study. H273Y has been associated with resistance to Peramivir while R374K has been associated with resistance to Oseltamivir, Zanamivir and Peramivir. Both substitutions are absent in the variants described in this study (<https://www.who.int/influenza/gisrs_laboratory/antiviral_susceptibility/NAI_Reduced_Susceptibility_Marker_Table_WHO.pdf?ua=1>).


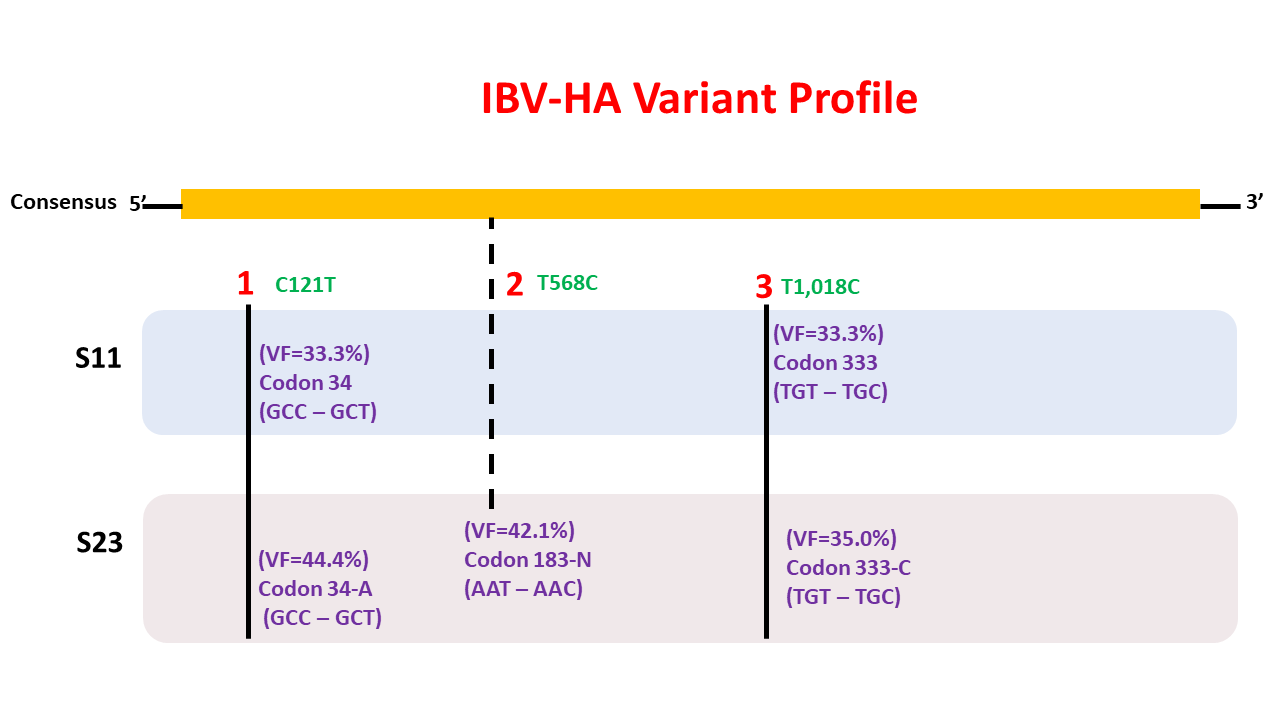


**Figure S7:** **Variant profile of IBV-HA samples 11 and 23.** Samples 11 and 23 share two variants C121T and T1018C. A third variant (T568C) distinguishes them from each other. Note that codons 34, 183 and 333 here are according to H1 numbering from first methionine. They would be codon 19, 169 and 319 in figure 3 where H1 is numbered not from first methionine but without signal peptide.


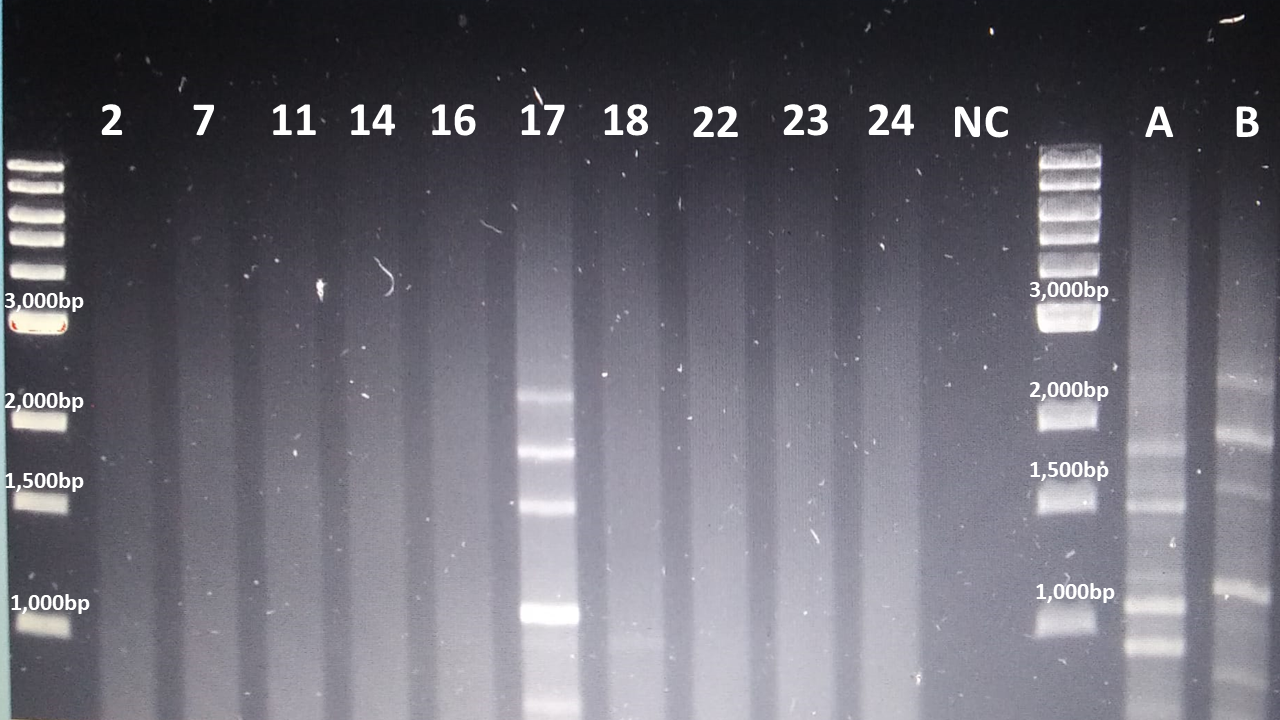


**Figure S8:** **Gel electrophoresis result of the FluB complete genome assay (Zhou et al., 2014).** All 10 samples from which complete HA and NA segments were recovered in this study (Table 1) were subjected to this assay. Lanes 1 and 13 contain molecular ladder. Lane 12 has negative control. Lanes 14 and 15 show the expected band patterns for IAV and IBV complete genome positive samples. Note that lane 15 has the positive control for this assay. Lane 14 has positive control for the IAV complete genome assay. It was loaded onto this gel only for comparison of the band patterns. Note sample 17 (lane 7) is the only sample positive for the assay. Please see Zhou et al., 2009 and 2014 for detailed protocols for the IAV and IBV complete genome amplification assays.
